# Construction and evaluation of an automated flow injection-stopped flow analyser for multipoint reaction rate spectrophotometric methods. Determination of ammonia nitrogen, creatinine and phosphate

**DOI:** 10.1155/S1463924691000342

**Published:** 1991

**Authors:** Constantinos A. Georgiou, Michael A. Koupparis

**Affiliations:** Laboratory of Analytical Chemistry Department of Chemistry University of Athens Panepistimiopolis, Kouponia Athens 15771 Greece

## Abstract

The construction and evaluation of a fully automated Flow Injection-Stopped Flow (FI-SF) spectrophotometric analyser is described. A microcomputer (Rockwell AIM 65) is used to control the analyser (sample injection, stop and start of the pump) through a suitable interface. Data acquisition is achieved using a 12 bit ADC card and a suitable subroutine in 6502 assembly language, allowing data sampling at a frequency of 7.5 kHz. The measurement interface and software were evaluated using a voltage ramp generator. A precision of 0.02-1.1% RSD (N =10) was obtained for voltage ramps in the range of 1-37 mVs^-1^. The FI-SF analyser was evaluated in routine analysis by developing FI-SF kinetic spectrophotometric methods for the determination of ammonia nitrogen (20-250 ppm, 0.4-2.5% RSD) based on the Berthelot reaction, creatinine (20-220 ppm, 0.9-3.6% RSD) based on the Jaffé reaction, and phosphate (5-30 ppm, 1.0-3.3% RSD) based on the phosphomolybdenum blue reaction. The reaction rate is measured by linear fitting of multiple absorbance readings vs time. Algorithms for automated estimation of the residence time, the linear range of the reaction curve, and data treatment are presented.


Journal of Automatic Chemistry, Vol. 13, No. 5 (September–October 1991), pp. 199–207

### Construction and evaluation of an automated flow injection-stopped flow analyser for multipoint reaction rate spectrophotometric methods. Determination of ammonia nitrogen, creatinine and phosphate

**Constantinos A. Georgiou and Michael A. Koupparis***

*Laboratory of Analytical Chemistry, Department of Chemistry, University of Athens, Panepistimiopolis, Kouponia, Athens 15771, Greece.*

*The construction and evaluation of a fully automated Flow Injection-Stopped Flow (FI-SF) spectrophotometric analyser is described. A microcomputer (Rockwell AIM 65) is used to control the analyser (sample injection, stop and start of the pump) through a suitable interface. Data acquisition is achieved using a 12 bit ADC card and a suitable subroutine in 6502 assembly language, allowing data sampling at a frequency of 7·5 kHz. The measurement interface and software were evaluated using a voltage ramp generator. A precision of 0·02–1·1% RSD (N = 10) was obtained for voltage ramps in the range of 1–37 mV $s^{-1}$. The FI-SF analyser was evaluated in routine analysis by developing FI-SF kinetic spectrophotometric methods for the determination of ammonia nitrogen (20–250 ppm, 0·4–2·5% RSD) based on the Berthelot reaction, creatinine (20–220 ppm, 0·9–3·6% RSD) based on the Jaffé reaction, and phosphate (5–30 ppm, 1·0–3·3% RSD) based on the phosphomolybdenum blue reaction. The reaction rate is measured by linear fitting of multiple absorbance readings vs time. Algorithms for automated estimation of the residence time, the linear range of the reaction curve, and data treatment are presented.*

### Introduction

Reaction rate (kinetic) methods have some advantages over equilibrium methods [1] and the automation is highly desirable in order to be used in routine analysis. Measurements of chemical systems evolving to equilibrium for analytical applications require the following modular units to perform specific tasks: (1) mixing the reactants; (2) monitoring the reaction progress (detection systems); (3) auxiliary electronics for signal conditioning and modification; and (4) computation and data handling. The precise mixing of reactants is one of the most important steps in kinetic procedures and its speed places a lower limit on the half-life of the reactions that can be employed in kinetic analysis. Automation of this step can be accomplished by: (*a*) automated batch mixing in conventional cells using magnetic or mechanical stirring, limited to slow reactions; (*b*) stopped-flow mixing, especially suitable for fast reactions; (*c*) continuous-flow mixing with air-segmented or unsegmented (flow-injection) sample processing, for reactions of intermediate rate; and (*d*) centrifugal mixing, for parallel fast determinations. Automated analysers based on the above approaches have been developed [1–3] varying in complexity, cost and capabilities.

The experimental data can be treated using three main approaches, i.e. the initial slope, fixed time, and variable time approaches. Flow injection (FI) in its traditional mode is, inherently, a kinetic fixed-time (one point) procedure, utilizing the signal (peak height) after a fixed time (residence time) [4]. Its greatest advantage over the stopped flow (SF) technique, the traditional approach for kinetic studies and measurements, is flexibility in reagent and sample handling, since more than two channels can be used for stepwise mixing of several incompatible reagents. On the other hand, the SF technique permits reaction rate kinetic measurements avoiding background signals. The marriage of the two approaches (FI and SF) resulted in the flow injection-stopped flow (FI-SF) technique, in which the sample zone is inserted in a flowing stream of reagent(s) and the reaction mixture is stopped in the detector cell allowing the monitoring of the reaction for a selected time interval. Restarting the flow effects the washing of the analyser's manifold from the previous mixture and the start of a new cycle [4,5]. FI-SF permits the increase of the reaction time without subsequent increase of the dispersion, allowing the implementation of rather slow reactions in FI systems, such as enzymic reactions. The mixing in FI-SF, in contrast to classical SF, is neither instant nor complete, restricting its use to reactions with half-lives more than several seconds. One further difference is that the hardware requirements for FI-SF are restricted to a pump capable of starting and stopping under control of a timer or a microcomputer synchronized with the injection unit of the system.

The FI-SF technique has been applied to the determination of $SO_2$ in wines [6,7] and boron in plants [8], and to the simultaneous determinations of magnesium and calcium [9]. This technique, in combination with the merging zones principle [5], is especially suitable for enzymic determinations, as background signals do not interfere and the consumption of enzyme reagents is

* To whom correspondence should be addressed.

0142–0453/91 $3.00 © 1991 Taylor & Francis Ltd.

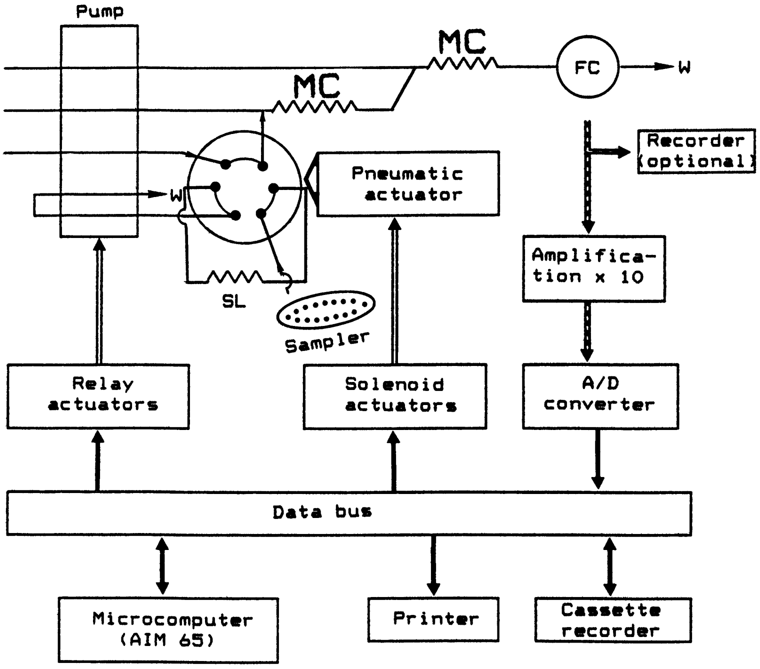

*Figure 1. Block diagram of the analyser configuration. Mixing coils (MC), sample loop (SL), flow cell (FC) and waste (W). Dashed, black and white lines for analog, digital and control signals respectively.*

minimized. In combination with electronic dilution [10], manual dilutions during sample preparation are avoided and the ratio enzyme/substrate is controlled. Using enzymic reactions, the substrates glucose in plasma [5] and urea [11], ethanol in blood and spirits [12], glycerol and triglycerides [13], and the activity of the enzymes galactic dehydrogenase [10], alkaline phosphatase, lipase, acetylcholinesterase and chymothripsin in plasma and serum [14], have been determined. FI-SF in conjunction with catalytic reactions has been used in the determination of various ions, such as Co(II) [15], cyanide [16], Cu(II) in foodstuffs [17], and Cu(II) and Hg(II) simultaneously [18]. Comparative studies revealed that in the determination of Cu(II) [17] many of the interferences appeared in a classical FI method, due to other metal ions, were minimized or eliminated in the FI-SF method. Most of the immunochemical methods that have been adapted to FI systems, such as for the determination of albumin in plasma [19], yeast mannan [20] and human serum immunoglobulin [21], use the FI-SF technique. FI-SF has also been used for infra-red spectrophotometric [22], voltammetric [23], and kinetic [24] studies.

The FI-SF instrumentation reported in previous papers was generally home made. In some, the Tecator commercial analyser was used [16–18]. Most of the systems used had no automation features incorporated and the flow was stopped with the use of a manual fluid switching valve [15,19,23,25]. In other systems, a timer controlled the pump and the kinetic curves were recorded and then were processed manually [5,6,11]. Microcomputers have been used in other systems to control the FI-SF system through digital I/O ports and to provide data logging through suitable interfaces. The kinetic approach used in these systems is the fixed time technique [7,9,10,12,16–18,20,21]. A variation of the fixed time approach utilizing the mean of absorbance pairs has also been described [13,14]. The fixed time approaches provide two point kinetic methods, therefore noise or random fluctuation during readings will cause significant error in the associated determinations. The development of an FI-SF analyser, described in this paper, featuring multipoint data acquisition and treatment will result in better

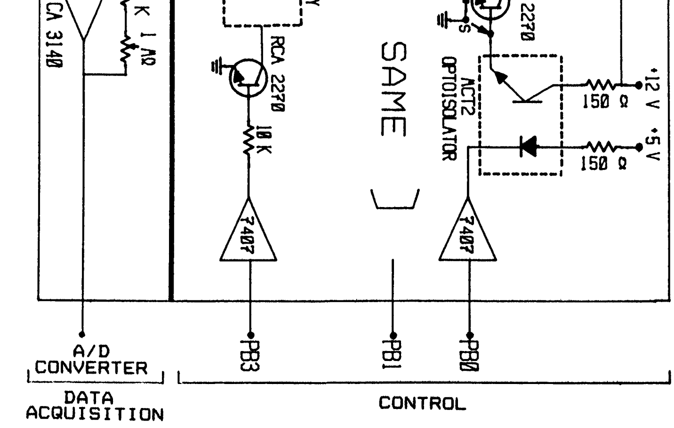

*Figure 2. Diagram of data acquisition and control interface. NO: normally open, NC: normally closed, S: manual switch, PB0, PB1 and PB2: bits 0, 1 and 2 of I/O port B of VIA 6522 chip.*

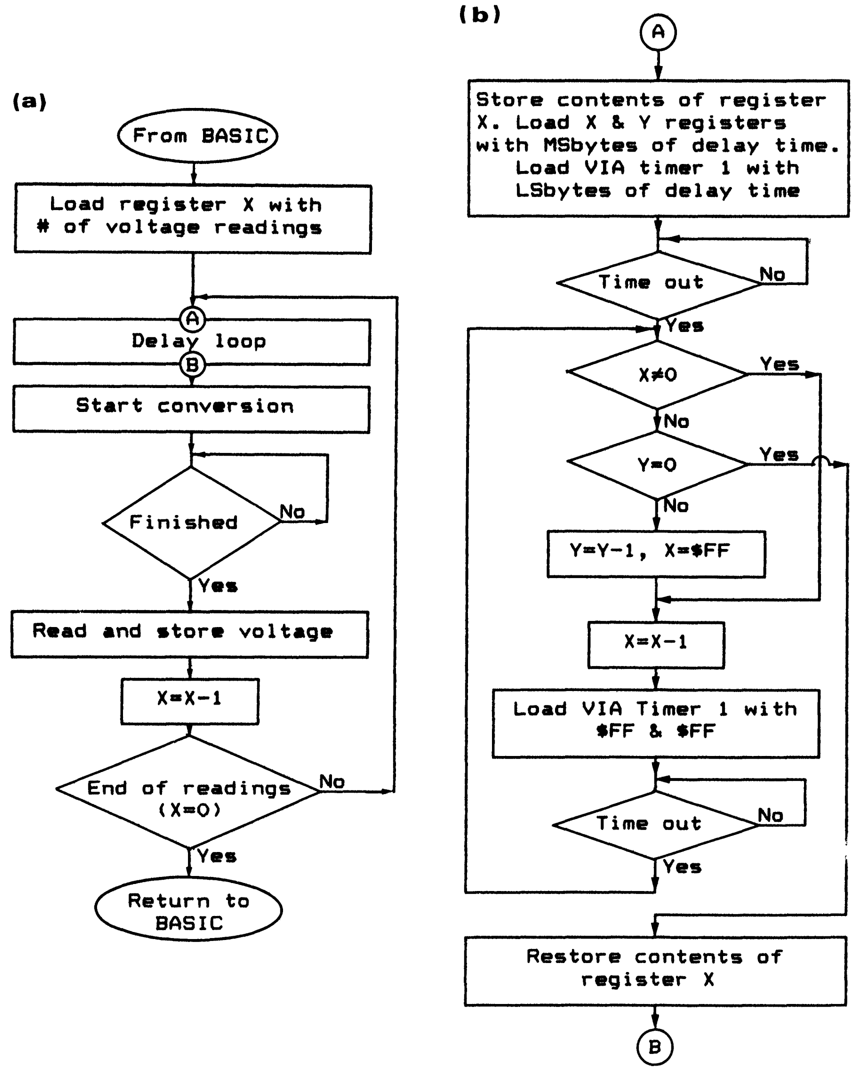

*Figure 3. Flow chart of the data logging routine: (a) conversion part; (b) delay loop.*

accuracy and precision. The fast data acquisition system (software and hardware) used extends the applicability of the described FI-SF analyser to relatively fast reactions.

### Experimental

### *Apparatus*

A diagram of the analyser configuration is shown in figure 1. It was constructed using the following components: (1) a remotely controllable peristaltic pump (Ismatec MPN-8); (2) a 6-port injection valve (Rheodyne 5001), equipped with a pneumatic actuator controlled by microcomputer through two 12 V DC solenoid valves (Angar 339-V); (3) mixing coils knitted [26] from 0·8 mm id Teflon tube; (4) a spectrophotometer (Bausch and Lomb, Spectronic 21), equipped with an 18 μl flow cell (Helma); (5) a microcomputer (Rockwell AIM 65), equipped with a BASIC interpreter and 6502 assembler—control of the system was achieved through the 8-bit parallel digital I/O ports (PA and PB) provided by the on board chip Versatile Interface Adapter (VIA) 6522 (this chip also incorporates two 16 bit timer-counters [T1 and T2], the external analogue-to-digital converter card used (Rockwell RM65-5302E) features the 12 bit successive approximation AD 574 (Analog Devices), and the typical conversion time was 35 μs, with a maximum value of 45 μs); (6) a sampler (Hook and Tucker, model A40) modified for microcomputer control; (7) a chart recorder (Heath-Schlumberger system) for the optional recording of absorbance during optimisation of the analytical methods—in this case the spectrophotometer's output (transmittance) is driven to the recorder through a logarithmic converter (Pacific Measurements, Inc., model 1002).

The voltage ramp generator used for the evaluation of the measurement system was constructed using an operational amplifier integrator with a 5 (±10%) MΩ resistor and a 10 (±10%) μF capacitor.

### *Reagents*

(*a*) *Ammonia nitrogen determination*: 0·20 M phenol solution, containing 0·038 M of potassium sodium tartrate. NaClO solution prepared daily with an 1 + 1 dilution of commercial bleach (~5% w/v in NaClO) with phosphate buffer 0·10 M, pH = 11·0. Nitrogen stock solution 1000 ppm prepared by dissolving the appropriate amount of primary standard grade ammonium sulphate. (*b*) *Creatinine determination*: 0·10 M HCl, 0·60 M NaOH and 0·030 M picric acid. Creatinine stock solution of 1000 ppm in 0·10 M HCl stored in the refrigerator when not in use. (*c*) *Phosphate determination*: Ammonium molybdate solution 20 g $l^{-1}$ (0·017 M) prepared a day before use in $H_2SO_4$ 0·10 M to ensure equilibrium of different forms of molybdenum in solution [27]. Ascorbic acid solution 10·6 g $l^{-1}$ (0.060 M) prepared daily. Phosphorus stock solution 100 ppm in P prepared by dissolving the appropriate amount of $KH_2PO_4$ in distilled water. (*d*) *Indicator stock solution*: $1{\cdot}0 \times 10^{-3}$ M of 2,6-dichlorophenol-indophenol dissolved in a bicarbonate buffer solution containing 210 mg $l^{-1}$ $NaHCO_3$. A working indicator solution of $1{\cdot}0 \times 10^{-4}$ M was prepared from the stock solution by dilution with bicarbonate buffer.

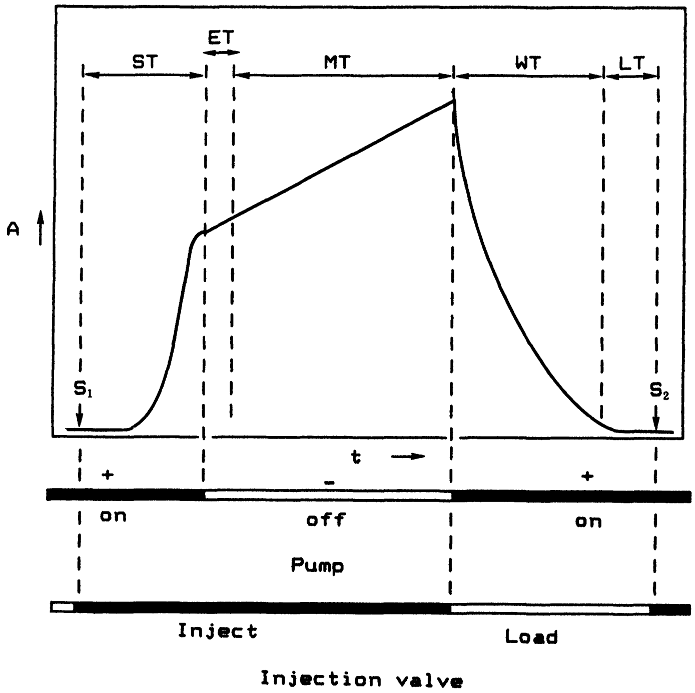

*Figure 4. Timing of FI-SF measurements (details in text).*

*Interface and associated software*

The diagram of the interface constructed for data acquisition and control is shown in figure 2. Details are given below.

*System control*

Loading and injecting the sample is accomplished by writing 0 to PB1 and PB0 addresses of the VIA chip, respectively. A manual override switch (S in figure 2) permits manual control of the injection valve. The flow stops by writing 0 to PB3, short circuiting the remote inputs 2, 3 and 5 of the pump.

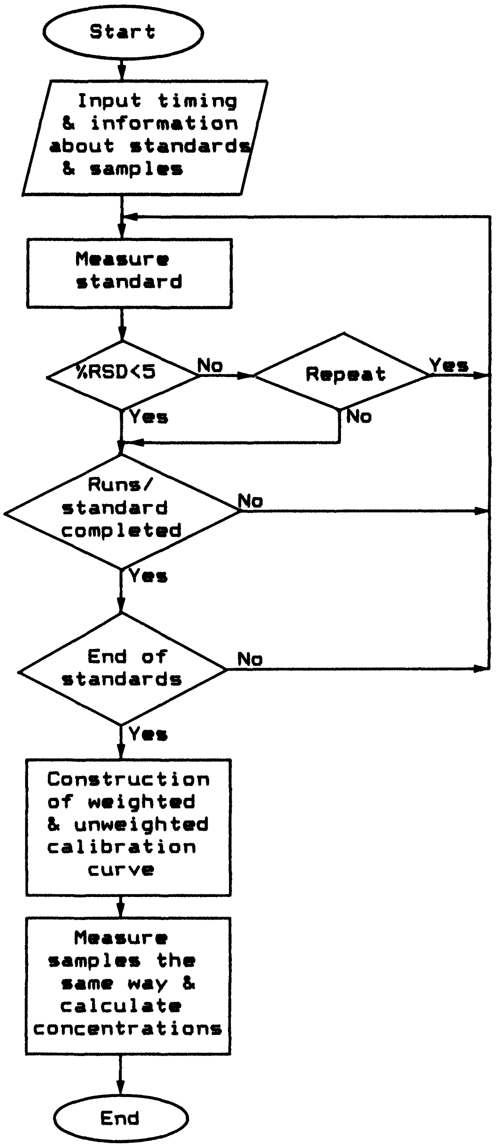

*Figure 5. Flow chart of the routine measurement program.*

*Data acquisition*

The output of the spectrophotometer destined for the chart recorder (0–1 V for 0–100% T) is fed to the A/D card after a ×10 amplification by a follower amplifier. The flow diagram of the data logging routine is given in figure 3. This routine, called LOG.OBJ was written in 6502 assembly to permit fast data acquisition through the A/D used and features a conversion part and a delay loop. The delay loop is based on two 8 bit software counters (registers X and Y) and on the VIA timer 1, which permits timing of high accuracy. In this way the use of an external hardware clock for long delay times is avoided. The data logging routine samples voltage (transmittance) readings equally spaced on the time axis and is callable from programmes written in BASIC after storing the number of readings (0–256) to be sampled in address $2DFF and the delay time sliced in four bytes in addresses $2DF0–$2DF3 (LSbyte-MSbyte) (mail box parameter passing technique). The A/D converter is polled until the end of conversion signal and the VIA timer 1 till time out. Calculating the overhead (89-100 μs)

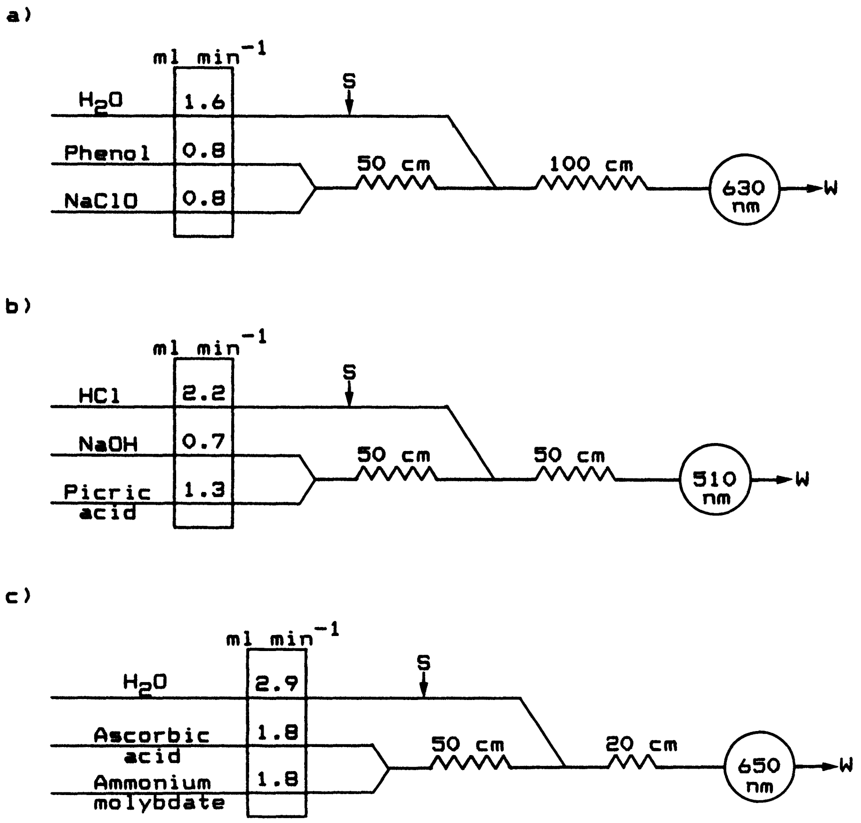

*Figure 6. Flow manifolds developed for the FI-SF determination of (a) ammonia nitrogen, (b) creatinine and (c) phosphate. Sample volume: 200 µl.*

*Table 1. Time parameters* (s) for the FI-SF methods for (1) ammonia nitrogen, (b) creatinine and (c) phosphate.*

| | ST | ET | MT | WT | LT | Total |
|---|---|---|---|---|---|---|
| a | 19·0 | 3·0 | 25·0 | 15·0 | 10·0 | 72·0 |
| b | 16·5 | 2·0 | 10·0 | 15·0 | 10·0 | 53·5 |
| c | 4·0 | 1·5 | 5·0 | 11·0 | 10·0 | 31·5 |

* Symbol meaning in text and figure 4.

introduced by this routine, the data acquisition rate is found to be one voltage reading every 124–134 µs (sampling frequency ~7·5 kHz).

### *Data treatment software*

Three programs have been developed in BASIC in order to optimize and run an FI-SF procedure. The first program destined for residence time calculation, called PEAK.BAS, injects a dye (2,6-dichlorophenolindophenol) solution in the flow manifold designed to be used for an FI-SF method and right after injection samples 256 absorbance readings for 120 s (one reading every 0·47 s). After the first rough calculation of the residence (peak) time, a second injection is done and 256 absorbance readings are again collected in a time span of ±10 s from the previously found residence time. The residence time is again calculated after smoothing the data points with a third degree polynomial filter using an 11 point box [28]. In this way the residence time is calculated with an accuracy of ±0·08 s. The residence time is the stop time required by the two other programs developed. Stopping the flow at the time of peak maximum results in improved sensitivity, but a rather limited linear range of the calibration curve [10].

The second program called INVQ.BAS, developed for the investigation of the optimum measurement time, calculates the linear range of the A = f(t) reaction curves. This program injects the most concentrated standard solution, for which the least linear range is expected, in the FI-SF manifold, and after stopping the flow at the residence time found, 256 absorbance readings are sampled for 120 s. Then, if the regression coefficient (r) of the A = f(t) curve is lower than a preset value (for example 0·999), the last point is dropped and the regression coefficient is calculated again. This procedure continues till the points remaining have the preset r value. The third program developed, called SLOPE.BAS, is used for routine measurements. The user is prompted to provide the number of standards, samples, runs per standard and sample, and the voltage readings to be sampled (3–256) during each measurement. Then the various time parameters for control of the analyser, according to figure 4, are requested. ST is the residence time found by the PEAK.BAS program, ET is the time required for mechanical equilibration before reading the voltage values, MT is the measurement time that is less or equal to the time found by the INVQ.BAS program, WT and LT are the wash and load time, respectively. The flow diagram of the SLOPE.BAS program is shown in figure 5. The standards are measured by calculation of the slope of the A = f(t) curve by linear regression of the

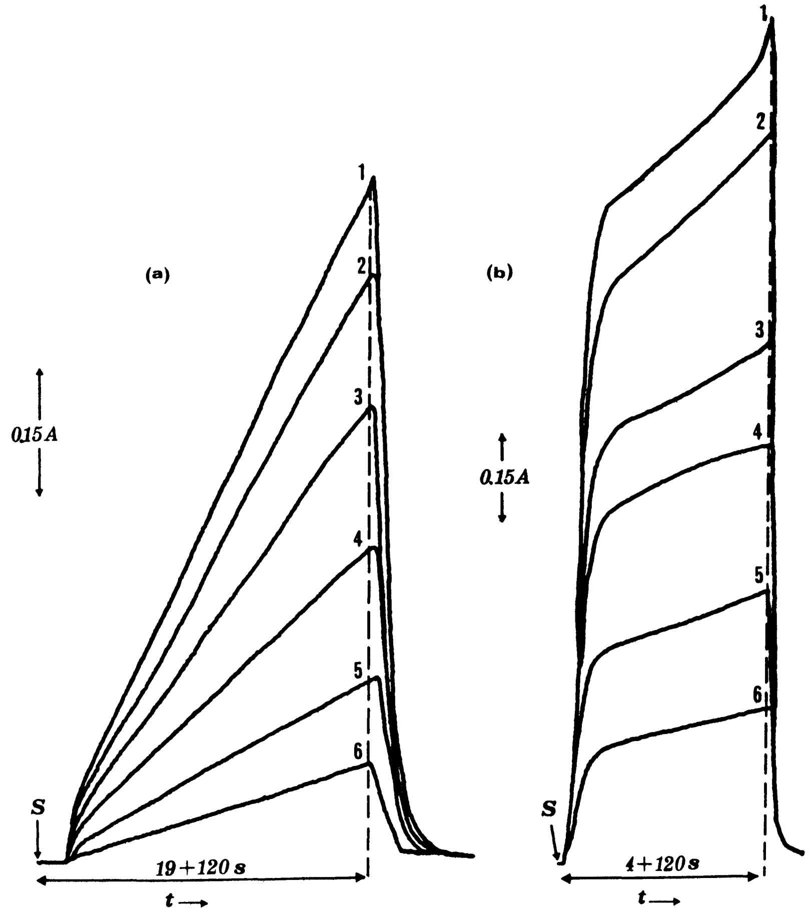

*Figure 7. Reaction curves obtained for the FI-SF determination of (a) ammonia nitrogen (1, 250; 2, 200; 3, 150; 4, 100; 5, 50; and 6, 20 ppm of nitrogen) and (b) phosphate (1, 30; 2, 25; 3, 20; 4, 15; 5, 10; and 6, 5 ppm phosphorus).*

*Table 2. Effect of the division of time between measurement (MT) and equilibration (ET) on the precision of reaction rate measurement in the phosphate determination.*

| MT/s | ET/s | %RSD (N = 10) |
|---|---|---|
| 5·0 | 0·0 | 2·9 |
| 4·5 | 0·5 | 2·4 |
| 4·0 | 1·0 | 2·0 |
| 3·5 | 1·5 | 1·1 |
| 3·0 | 2·0 | 1·0 |
| 2·5 | 2·5 | 1·1 |

absorbance readings sampled during MT. The program displays and prints this value, along with its SD and %RSD (within run parameters showing the linearity of the reaction curve). If the %RSD value exceeds 5 the program asks for rejection of the value found and performs another run. When replicate measurements per standard are requested, the procedure is repeated and afterwards the mean slope value and the between run SD and %RSD are calculated and displayed. Then the program calculates the calibration curve by linear regression using the mean slope values of all standards. If three or more runs per standard are requested, the program also calculates and displays the weighted linear regression curve using as weighting factor for each point is normalized inverse variance [29]. The samples are then run in the same way and the concentration values (resulting from the two calibration curves) along with pertinent statistics (SDs and %RSDs) are calculated and displayed.

### *Determination of ammonia nitrogen, creatinine and phosphate*

The flow manifolds designed for the FI-SF determination of these three analytes are shown in figure 6. Ammonia nitrogen determination is based on the Berthelot reaction, in which ammonia reacts with hypochlorite to form chloramine, which, in turn, reacts with phenol under alkaline conditions to form indophenol that is monitored at 630 nm. Creatinine is determined according to the Jaffé reaction, in which a coloured complex ($\lambda_{max}$ = 510 nm) is formed during its reaction with picrate in alkaline solution. Creatinine sample solutions are injected in a 0·10 M HCl stream for better pH control (the reaction takes place at a [NaOH] ~ 0·04 M after the streams merge). The kinetic determination of phosphate is based on the rate of the reduction of molybdophosphoric acid by ascorbic acid. The formation of the phosphomolybdenum blue product is followed at 650 nm.

The residence time of each manifold was estimated by the PEAK.BAS program by injection of 200 μl of the working indicator (2,6-dichlorophenolindophenol) solution in the $NaHCO_3$ buffer pumped through the manifolds used. The absorbance is monitored at 522 nm (the isosbestic

*Table 3. Calibration curves for the FI-SF determination of: (a) ammonia nitrogen, (b) creatinine and (c) phosphate.*

| | Slope mA s$^{-1}$ ppm$^{-1}$ | Intercept | Linear range/ppm | r | %RSD (N = 6) |
|---|---|---|---|---|---|
| a | 0·0237 ± 0·0005 | 0·48 ± 0·08 | 20–250 | 0·9990 | 0·4–2·5 |
| b | 0·0356 ± 0·0003 | −0·005 ± 0·040 | 20–220 | 0·9998 | 0·9–3·6 |
| c | 2·55 ± 0·01 | 1·0 ± 0·2 | 5–30 | 0·99993 | 1·0–3·3 |

*Table 4. Results of the analysis of synthetic samples.*

| Taken | Found ± SD (N = 10) | % Relative error |
|---|---|---|
| Nitrogen concentration (ppm) | | |
| 200 | 199·2 ± 0·8 | −0·4 |
| 150 | 151 ± 1 | +0·7 |
| 20·0 | 20·8 ± 0·5 | 4·0 |
| | 20·2 ± 0·5* | 1·0 |
| Creatinine concentration (ppm) | | |
| 220 | 216 ± 6 | −1·8 |
| 140 | 143 ± 3 | 2·1 |
| 20·0 | 19·0 ± 0·7 | −5·0 |
| | 19·7 ± 0·7* | −1·5 |
| Phosphorus concentration (ppm) | | |
| 30·0 | 29·9 ± 0·2 | −0·3 |
| 15·0 | 15·2 ± 0·3 | 1·3 |
| 5·0 | 4·9 ± 0·2 | −2·0 |
| | 5·0 ± 0·2* | 0·0 |

* Results obtained using the weighted regression calibration curve.

wavelength of the indicator). Subsequently, the linear range of the A = f(t) reaction curves are calculated running the INVQ.BAS program and then the SLOPE.-BAS programme is used for the routine measurements. The timing parameters for the three determinations are presented in table 1.

During measurement the reservoirs of the reagents were immersed in a 25 °C water bath.

### Results and discussion

The FI-SF analyser was evaluated using simulated reaction rate curves (ramps) generated by an analogue integrator and with real chemical kinetic determinations.

### *Simulated reactions*

An analogue integrator with an RC time constant of 50 s was utilized to generate highly reproducible voltage ramps. The slope of these ramps was varied in the range of 1–37 mV s$^{-1}$ by changing the input voltage ($V_{in}$) using a reference voltage source. The simulated reaction curves were monitored by sampling 100 voltage readings for a MT of 23 s. The simulated calibration curves (mV s$^{-1}$ vs $V_{in}$) were very linear (r > 0·9999997) with between run precision of 0·02–1·1 %RSD ($N$ = 10). Simulated standard 'samples' could be determined with relative errors varying from 0·01 to 0·7%. The upper limit of the slope range (37 mV s$^{-1}$) can be extended to higher values using shorter measurement times (MT < 23 s). These results show the excellent operation of the measurement interface and software while monitoring a changing voltage.

### *Determination of ammonia nitrogen, creatinine and phosphate*

In order to evaluate the FI-SF analyser in kinetic determinations the aforementioned three kinetic methods, already used with the SF approach [30–32], were selected. From the reaction rate point of view the Berthelot reaction can be considered as relatively slow, the Jaffé reaction as of intermediate rate and the phosphomolybdenum blue reaction as a rapid one. The latter two have also been adapted to the traditional FI technique (peak height measurement) [33,34]. These determinations require more than one reagent solution to be added to the sample solution, therefore in the classical SF approach, in which only two channels are available, a time consuming and tedious precise premixing of the sample with one of the reagents is required [30–32]. In the FI-SF manifolds designed (see figure 6) the otherwise unstable mixed reagents (phenol + NaClO and ascorbic acid + molybdate) are prepared *in situ* in the first mixing coil (50 cm). The second mixing coil, used for sufficient mixing of the sample solutions with the mixed reagents, has a length (and consequently stop time (ST) and equilibration time (ET) (table 1)) related to the reaction rate, so that the reaction almost starts in the cell. The design of these FI-SF schemes (reagent concentrations, flow rates, etc.) was based on information available from pertinent literature [27, 30–32] and optimized in several preliminary experiments.

Typical reaction curves obtained for ammonia and phosphate determinations during the investigation study are shown in figure 7. The reaction curves for creatinine were similar to those obtained for ammonia. For ammonia and creatinine the A versus t curves were linear for at least 120 s for all the standards used. Measurement times (MT) of 25 and 10 s, respectively (table 1), were selected as a compromise between precision and measurement throughput. For the phosphate determination (figure 7[b]) the curves have two distinct linear parts due to the reaction mechanism [30]. In the FI-SF determination the absorbance was monitored during the first stage, maximizing sensitivity and minimizing the analysis time. From the at least 10 s linear part of the reaction curve, 1·5 s is used for equilibration (ET) after the flow stop and 5·0 s for measurement (MT).

Dividing the time between equilibration (ET) and measurement (MT) is very critical for the precision in the case of a limited linear range of the reaction curve, as in the phosphate determination. Increasing the equilibration time the precision increases, up to a plateau, even if the measurement time decreases (table 2). All the timing parameters, along with the total time for a measurement, for the three determinations are summarized in table 1.

Typical calibration curves for the kinetic FI-SF determination of the three analytes are shown in table 3. The linearity is good ($r > 0{\cdot}999$) and the between run RSD varies from 0·4–3·6%. The measurement throughput was (table 1) 50, 67 and 114 per hour for the determination of ammonia nitrogen, creatinine and phosphate, respectively. Synthetic samples were analysed and the results are shown in table 4. Relative errors ranged from 0·3 to 5% using the unweighted regression curve for calibration. More accurate results were obtained, especially for low concentrations, if the weighted regression curve is used for calibration. However, in this case a higher number of runs per standard is required to obtain the variance of each standard, resulting in a lower sample throughput.

### Conclusions

The results obtained indicate the suitability of the FI-SF analyser to perform multipoint spectrophotometric kinetic determinations in the routine analysis. In comparison with the classical stopped-flow (SF) analyser [32] the FI-SF analyser has the following features: (1) It has the flexibility of using more than two channels, so that multistep analytical schemes can be used in automated kinetic analysis, unstable mixed reagents are prepared just before use on line, and the sample solution is injected directly without any premixing with a reagent (very common in SF). (2) The measurement throughput (50–114 per hour for the described determinations) is comparable with that of SF for slow and intermediate rate reactions. (3) Since in FI-SF the mixing of reactants is effected by the flow in coils and an equilibration time is required before measurement, very fast reactions cannot be utilized and in such cases SF remains the approach of choice. Phosphorus determination (requiring 10·5 s for stop, equilibration and measurement) seems to be at around the limiting time for the FI-SF system.

In comparison with the traditional FI technique, FI-SF has the advantages of (*a*) multipoint measurements resulting in increased precision and accuracy; (*b*) correction of background signals (for example spectrophotometric determinations in coloured sample solutions); (*c*) performing differential kinetic determinations in mixtures, and (*d*) application of slow reactions in the FI technique.

The interface and the data logging routine described in this work can also be used in peak height, peak width (FI pseudo-titrations) and other FI gradient techniques provided that suitable data treatment software is written. The use of other detection systems (for example ISEs) can be accomplished with minimal changes in the interface to provide the appropriate signal conditioning.

### Acknowledgements

We gratefully acknowledge support from the Ministry of Industry, Energy and Technology, General Secretariat of Research and Technology of Greece, the Greek State Scholarships Foundation and the Greek National Drug Organization.

### References

1. Mottola, H. A., *Kinetic Aspects of Analytical Chemistry.* John Wiley and Sons, New York (1988).
2. Malmstadt, H. V., Krottinger, D. L. and McCracken, M. S., in Foreman, J. K. and Stockwell, P. B., *Topics in Automatic Chemical Analysis 1.* Ellis Horwood Ltd, Chichester (1979), p. 95.
3. Valcarcel, M. and Luque de Castro, M. D., *Automatic Methods of Analysis.* Elsevier, Amsterdam (1988).
4. Ruzicka, J. and Hansen, E. H., *Flow Injection Analysis.* 2nd Edition, John Wiley and Sons, New York (1988).
5. Ruzicka, J. and Hansen, E. H., *Analytica Chimica Acta,* **106** (1979), 207.
6. Ruzicka, J. and Hansen, E. H., *Analytica Chimica Acta,* **114** (1980), 19.
7. Lazaro, F., Luque de Castro, M. D. and Valcarcel, M., *Analytical Chemistry,* **59** (1987), 950.
8. Arruda, M. A. Z. and Zagatto, E. A. G., *Analytica Chimica Acta,* **199** (1987), 137.
9. Kagenow, H. and Jensen, A., *Analytica Chimica Acta,* **145** (1983), 125.
10. Olsen, S., Ruzicka, J. and Hansen, E. H., *Analytica Chimica Acta,* **136** (1982), 101.
11. Ramsing, A., Ruzicka, J. and Hansen, E. H., *Analytica Chimica Acta,* **114** (1980), 165.
12. Worsfold, P. J., Ruzicka, J. and Hansen, E. H., *Analyst,* **106** (1981), 1309.
13. Masoom, M. and Worsfold, P. J., *Analytica Chimica Acta,* **188** (1986), 281.
14. Masoom, M. and Worsfold, P. J., *Analytica Chimica Acta,* **179** (1986), 217.
15. Yamane, T., *Analytica Chimica Acta,* **130** (1981), 65.
16. Linares, P., Luque de Castro, M. D. and Valcarcel, M., *Analytica Chimica Acta,* **161** (1984), 257.
17. Lazaro, F., Luque de Castro, M. D. and Valcarcel, M., *Analytica Chimica Acta,* **165** (1984), 177.
18. Lazaro, F., Luque de Castro, M. D. and Valcarcel, M., *Fresenius' Z. Anal. Chem.,* **320** (1985), 128.
19. Lim, C. S., Miller, J. N. and Bridges, J. W., *Analytica Chimica Acta,* **114** (1980), 183.
20. Worsfold, P. J. and Hughes, A., *Analyst,* **109** (1984), 339.
21. Worsfold, P. J., Hughes, A. and Mowthorpe, D. J., *Analyst,* **110** (1985), 1303.
22. Morgan, D. K., Danielson, N. D. and Katon, J. E., *Analytical Letters,* **18** (1985), 1979.
23. Tougas, T. P. and Curran, D. J., *Analytica Chimica Acta,* **161** (1984), 325.
24. Hungerford, J. M., Christian, G. D., Ruzicka, J. and Giddings, J. C., *Analytical Chemistry,* **57** (1985), 1794.
25. Yoza, N. Kurokawa, Y., Hirai, Y. and Ohashi, S., *Analytica Chimica Acta,* **121** (1980), 281.
26. Selavka, C. M., Jiao, K. S. and Krull, I. S., *Analytical Chemistry,* **59** (1987), 2221.
27. Crouch, S. R. and Malmstadt, H. V., *Analytical Chemistry,* **39** (1967), 1084.
28. Savitzky, A. and Golay, M. J. E., *Analytical Chemistry,* **36** (1964), 1627.

29. Miller, J. C. and Miller, J. N., *Statistics for Analytical Chemistry*. Ellis Horwood, Chichester (1986).

30. McCracken, M. S. and Malmstadt, H. V., *Talanta*, **26** (1979), 467.

31. McCracken, M. S. and Malmstadt, H. V., *J. Assoc. Off. Anal. Chem.*, **62** (1979), 23.

32. Koupparis, M. A., Walczak, K. M. and Malmstadt, H. V., *Journal of Automatic Chemistry*, **2(2)** (1980), 66.

33. van Staden, J. F., *Fresenius' Zeitschrift für Analytische Chemie*, **315** (1983), 141.

34. Hansen, E. H. and Ruzicka, J., *Journal of Chemical Education*, **56** (1979), 677.

